# Near-Death High-Frequency Hyper-Synchronization in the Rat Hippocampus

**DOI:** 10.3389/fnins.2019.00800

**Published:** 2019-07-31

**Authors:** Yujiao Zhang, Zhenyi Li, Jing Zhang, Zongya Zhao, Hongxing Zhang, Martin Vreugdenhil, Chengbiao Lu

**Affiliations:** ^1^School of Psychology, Xinxiang Medical University, Xinxiang, China; ^2^International-Joint Lab for Non-Invasive Neural Modulation of Henan Province, Department of Neurobiology and Physiology, Xinxiang Medical University, Xinxiang, China; ^3^School of Biomedical Engineering, Xinxiang Medical University, Xinxiang, China; ^4^Department of Life Sciences, School of Health Sciences, Birmingham City University, Birmingham, United Kingdom

**Keywords:** hippocampus, gamma oscillations, chloral hydrate, beta oscillations, theta oscillations

## Abstract

Near-death experiences (NDE) are episodes of enhanced perception with impending death, which have been associated with increased high-frequency (13–100 Hz) synchronization of neuronal activity, which is implicated in cognitive processes like perception, attention and memory. To test whether the NDE-associated high-frequency oscillations surge is related to cardiac arrest, recordings were made from the hippocampus of anesthetized rats dying from an overdose of the sedative chloral hydrate (CH). At a lethal dose, CH caused a surge in beta band power in CA3 and CA1 and a surge in gamma band power in CA1. CH increased the inter-regional coherence of high-frequency oscillations within and between hippocampi. Whereas the surge in beta power developed at non-lethal chloral hydrate doses, the surge in gamma power was specific for impending death. In contrast, CH strongly suppressed theta band power in both CA1 and CA3 and reduced inter-regional coherence in the theta band. The simultaneously recorded electrocardiogram showed a small decrease in heart rate but no change in waveform during the high-frequency oscillation surge, with cardiac arrest only developing after the cessation of breathing and collapse of all oscillatory activity. These results demonstrate that the high-frequency oscillation surge just before death is not limited to cardiac arrest and that especially the increase in gamma synchronization in CA1 may contribute to NDE observed both with and without cardiac arrest.

## Introduction

Near-death experiences (NDE) are “intense psychological experience of debated nature, characterized by an atypical state of consciousness occurring during an episode of apparent unconsciousness and usually in life-threatening conditions” (Palmieri et al., 2014). NDE have been reported by successfully resuscitated cardiac arrest patients where the NDE occurred during cerebral hypoxia (French, 2005) and patients where life support was discontinued (Auyong et al., 2010). Hallucinations and memory flashbacks in NDEs suggest the involvement of limbic structures like the hippocampus (French, 2005) and the temporal lobe (Vanhaudenhuyse et al., 2009), with oscillatory activity of NDE memories very similar to real life memory recall (Palmieri et al., 2014). During NDE’s, EEG change transiently to that similar to a conscious state (Auyong et al., 2010), with a period of a few minutes of increased high-frequency activity (Chawla et al., 2017). In anesthetized rats killed by high potassium-induced cardiac arrest a similar increase in gamma band (γ, 30–100 Hz) frequency oscillations was reported, which was suggested to reflect a heightened state of conscious awareness (Borjigin et al., 2013).

Synchronization of neuronal activity takes place in various brain areas in different frequency bands: theta (θ: 3–8 Hz), alpha (α: 8–13 Hz, most prominent in the visual cortex), beta (β: 13–30 Hz) and γ. These neural oscillations provide temporal frame for the information processing related to the perception and memory (Mizuseki et al., 2009; Amemiya and Redish, 2018). Synchronization of neuronal activity in the γ band has been implicated in perception, attention, encoding and retrieval of memory (Lisman and Jensen, 2013; Hanslmayr et al., 2016) and dynamic routing of information (Colgin et al., 2009). β oscillations have been implicated in top-down processing, long-range communication and interactions between attentional and emotional systems (Fries, 2015; Marco-Pallares et al., 2015; Spitzer and Haegens, 2017). θ oscillations regulate γ activity, coordinate communication between brain regions and are involved in sensory as well as memory processes (Colgin et al., 2009; Lisman and Jensen, 2013). The careful interplay of θ, β and γ rhythms is necessary for effective, precise and selective neuronal communication (Fries, 2015) and therefore, disruption of oscillatory activities can lead to aberrant cognitive function. Indeed, loss of consciousness, e.g., under isoflurane anesthesia is associated with disruption of high-frequency (>13 Hz) oscillations and inter-regional coherence (Imas et al., 2005, 2006) and sedation by barbiturates is linked to increase in β oscillation power (Akeju and Brown, 2017).

An anecdotal observation of a surge in high-frequency oscillation power in the rat hippocampus area CA1 during euthanasia by an overdose of the barbiturate pentobarbital (unpublished observation, MV), led to the question whether the high-frequency oscillation surge occurs during euthanasia by overdose with other drugs.

Chloral hydrate (CH), a halogenated hydrocarbon diol, is a sedative-hypnotic drug (Avlonitou et al., 2011; Sezer and Alehan, 2013) and the mechanism of action of its active metabolite, 2,2,2-trichloroethanol, is similar to that of barbiturates (Peoples and Weight, 1994). CH has since 1869 been in use for hypnotic or sedative purposes in low dose (40–80 mg/kg) (Avlonitou et al., 2011), but is fatal at higher doses.

To test whether the increase in high-frequency oscillations induced by cardiac arrest (Borjigin et al., 2013) is dependent on the method of death, we examined whether similar EEG changes would happen during death by an overdose of CH.

In this study we found that death induced by an overdose of CH was associated with hyper-synchronization in beta/gamma bands of hippocampal CA1 and CA3 areas, just before cardiovascular death, confirming that the high-frequency oscillation surge is not dependent on method of death and may well underlie NDEs.

## Materials and Methods

### Animals

In all studies, the Wistar rats were purchased from Beijing Vital River Laboratory Animal Technology Co., Ltd., which were acclimatized in our animal facility for at least 1 week before experiments. The experimental procedures were approved by Xinxiang Medical University Committee on Use and Care of Animals. All experiments were conducted using adult male rats (370–470 g). The rats were maintained on a light: dark cycle of 12:12 h and provided with *ad libitum* food and water.

### General Anesthesia and Local Field Potential Recording

In order to record oscillatory activity of local field potentials in the hippocampus, electrodes were implanted in rats kept under stable urethane/medetomidine anesthesia. In order to observe the changes in oscillatory activity associated with death by a drug overdose, CH was injected IP after oscillatory activity was stabilized.

Bipolar twisted wire recording electrodes were made using 50 μm Formvar insulated 80% nickel, 20% chromium wire (Advent Research Materials, Oxford, United Kingdom) with 0.5 mm distance between the recording tips.

All rats were initially anesthetized by an intraperitoneal injection of 1.2 g/kg urethane. A 0.04 mg/kg medetomidine bolus injection was administered 1 h after the urethane injection, before attaching a subcutaneous drip filled with 0.04 mg/kg/h medetomidine and 5 mg/kg/h urethane in sterile saline. This provided a deep anesthesia, sufficient for surgery.

After exposure of the skull, 0.8 mm diameter holes were drilled in the skull and the dura was opened, to place recording electrodes bilaterally in CA1: AP 4.2, ML 2.0 (relative to bregma), DV (from cortex surface) 2.2 and in CA3: AP 3.2, ML 2.8, DV (from cortex surface) 3.4. A (skull screw) reference electrode was placed at AP 10.5, ML 0.0. After completion of surgery, the medetomidine/urethane drip speed was decreased to obtain a light, but sufficient level of anesthesia, titrated to individual rat’s needs, which was monitored by regular pedal withdrawal checks. The rat temperature was kept at 37°C using a rectal probe feedback-operated DC heating pad (ATC1000, World Precision Instruments). Recordings were made relative to reference with a RHD2132 16-channel digital amplifier controlled by a RHD2000 interface board and RHD2000 interface software (Intan Technologies, Los Angeles, CA, United States). Recordings were band-pass filtered at 0.5 Hz–500 Hz and then sampled at 2 kHz using a CED power-1401 (Cambridge Electronic Design, Cambridge, United Kingdom) controlled by Spike-2 software (Cambridge Electronic Design).

In some experiments the electrocardiogram (ECG) was recorded simultaneously with local field potentials, using clip electrodes on both front paws. In some experiments a video recording was made of the rat to monitor the breathing rate.

### Spectral Analysis

Spectral analysis was done using Spike-2 software (Cambridge Electronic Design). Local field potential recordings were first inspected artifacts and recordings were omitted 5 s before and 5 s after artifacts. The oscillation power was calculated from 30 s unfiltered recording epoch by fast Fourier transform (1 Hz bin size, Hanning window). Heart rate (HR) was calculated from QRS peak intervals in the ECG and averaged over 1 s. ECG waveforms were generated by marking the QRS peaks in the ECG and zeroing all cardiac cycles over 30 s on the QRS peak mark. Breathing rate was determined from inspection of video footage of the rat.

### Coherence Analysis

Coherence (COH) analysis estimates the degree of synchronization between two oscillations. To calculate the inter-regional coherence, data was pre-processed with Spike-2 software and then imported to Matlab (Mathworks, Natic, MA, United States). The HERMES toolbox was used for calculating the coherence (Niso et al., 2013), which measures the linear correlation between two variables *x*(*t*) and *y*(*t*) as a function of the frequency, f. It is the squared module of the coherency function (K), which is the ratio between the cross power spectral density, *S*_*xy*_(*f*), between *x*(*t*) and *y*(*t*), and their individual power spectral densities *S*_*xx*_(*f*) and *S*_*yy*_(*f*):

$$K_{x\,y}\,\left(f\right)=\frac{S_{x\,y}\,\left(f\right)}{\sqrt{S_{x\,x}\,\left(f\right)\,S_{y\,y}\,\left(f\right)}}$$

Thus, the coherence is defined as:

$$C\,O\,H_{x\,y}\,\left(f\right)={\left|K_{x\,y}\,\left(f\right)\right|}^{2}=\frac{{\left|S_{x\,y}\,\left(f\right)\right|}^{2}}{S_{x\,x}\,\left(f\right)\,S_{y\,y}\,\left(f\right)}$$

COH values were normalized to fit in the range between 0 and 1 [range: 0 ≤ COH_*xy*_ (f) ≤ 1]. Zero (0) means no linear dependence between *x*(*t*) and *y*(*t*) at frequency f. One (1) means correspondence between *x*(*t*) and *y*(*t*) at frequency f.

### Statistics

Data analysis was carried out using SPSS software (SPSS Inc., Chicago, IL, United States). Where one or both data sets were not normally distributed within-group comparisons were performed using the Wilcoxon signed rank test, else a paired Student’s *t*-test was used. A one-way ANOVA was used to test the dose-dependence of effects. Averaged results were expressed as mean ± standard error of the mean (s.e.m.). Effects were considered significant if *P* < 0.05.

## Results

### Baseline Oscillations

The medetomidine dose was titrated such that anesthesia was just sufficient to block pedal reflex. Local field potentials were recorded bi-laterally in area CA1 and in CA3 of the right dorsal hippocampus of anesthetized rats. The bipolar electrodes were aimed to straddle the stratum pyramidale and the subtraction of the recordings from the short and long electrode focus on the locally generated oscillations. After 20–30 min, oscillatory activity stabilized.

**FIGURE 1:**
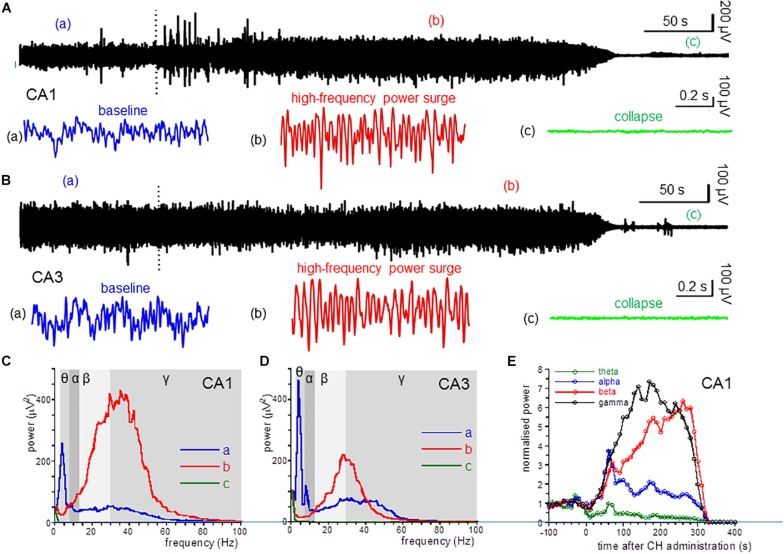
CH-induced changes in oscillatory power. **(A)** Local field potential recordings from the dorsal hippocampus area CA1, before and after application of a lethal dose of CH (650 mg/kg, dotted line). Inserts give a 1-s example from baseline (**a:** blue trace), during the CH-induced surge in high-frequency oscillations (**b:** red trace) and after collapse of oscillatory activity (**c:** green trace). **(B)** Local field recording from area CA3. Details as in **(A)**. **(C)** Power spectra of recording in **(A)** at baseline (**a:** red line), during the CH-induced surge in high-frequency oscillations (**b:** red trace) and after collapse of oscillatory activity (**c:** green trace). **(D)** Power spectra of recording in **(B)**. Details as in **(C)**. **(E)** Normalized (to the average of the 5 min before CH administration) power in the theta (θ: 3–8 Hz; green line) band, alpha (α: 8–13 Hz; blue line) band, beta (β: 13–30 Hz; red line) band, and low gamma (γ: 30–55 Hz; black line) band, as function of time after CH administration for the recording in **(A)**.

The local field potential recorded in CA1 showed oscillatory activity (example in Figure 1Aa) that was analyzed by fast Fourier transforms. Figure 1C (blue line) shows the power spectrum for the recording in Figure 1Aa. Average power within the different frequency bands was used to quantify effects of CH. For 14 CA1 recordings, the average power was 36 ± 11 μV^2^ for the θ band, 14 ± 4 μV^2^ for the α band, 14 ± 4 μV^2^ in the β band and 4.3 ± 1.2 μV^2^ in the γ band. Peak power in the high-frequency (>13 Hz) band was at a peak frequency of 21.1 ± 1.8 Hz. Figure 1Ba gives an example of oscillatory activity recorded in area CA3. For CA3, θ power was 52 ± 41 μV^2^, α power was 16 ± 9 μV^2^, β power was 16 ± 9 μV^2^ γ power was 8.1 ± 4.6 μV^2^. In recordings from rats with a discernible high-frequency oscillation power peak the peak frequency was 39.2 ± 6.6 Hz.

### CH Causes a High-Frequency Oscillation Surge in Area CA1 and CA3

Administration of CH at 650 mg/kg, IP caused the death in all rats tested. This CH overdose caused a rapid and dramatic increase of β power and γ power in area CA1. In contrast, CH caused a strong suppression of θ power [example in Figures 1Ab,C (red line)]. Figure 1E shows the development of the oscillation power with time after CH application. β power increase and θ power decrease usually developed slower than the γ power increase. The high-frequency oscillation surge lasted for 1–3 min, after which oscillatory power in all frequency bands suddenly sharply reduced and, within tens of seconds, all oscillatory activity ceased. This collapse of activity occurred in all locations simultaneously (examples in Figure 1). The 30 s just before the collapse of oscillatory power was taken to quantify the CH-induced changes.

Compared to the average oscillation power in the 5 min before administration (baseline), CH increased β power in CA1 by 205 ± 51% [*Z*_(13)_ = −3.18, *P* = 0.001] and increased γ power by 140 ± 62% [*Z*_(13)_ = −3.11, *P* = 0.002]. In contrast CH decreased θ power by 58 ± 9% [*Z*_(13)_ = −3.11, *P* = 0.002]. CH did not affect α power [127 ± 14% of baseline, *Z*_(13)_ = −0.94, *P* = 0.345] (Table 1). CH reduced the high-frequency power peak to 27.7 ± 1.7 Hz [*t*_(13)_ = −3.25, *P* = 0.006].

**TABLE 1:** Change in average power in the theta (3–8 Hz), alpha (8–13 Hz), beta (13–30 Hz), and gamma (30–100 Hz) bands for different CH doses.

**Dose**	**n**	**theta**	**alpha**	**beta**	**gamma**
100 mg/kg	8	82 ± 10	93 ± 7	123 ± 6^∗^	106 ± 6
300 mg/kg	8	30 ± 4^∗^	105 ± 24	176 ± 24^∗^	72 ± 5^∗^
650 mg/kg	14	42 ± 9^∗∗^	126 ± 14	305 ± 51^∗∗^	241 ± 62^∗^

*The 30 s before oscillatory activity collapse (for 650 mg/kg) or the plateau measure after CH administration (for 100 and 300 mg/kg) are expressed as% of baseline. Data are mean ± s.e.m., Wilcoxon signed rank test probability is indicated as ^∗^*P* < 0.05 and ^∗∗^*P* < 0.01.*

**FIGURE 2:**
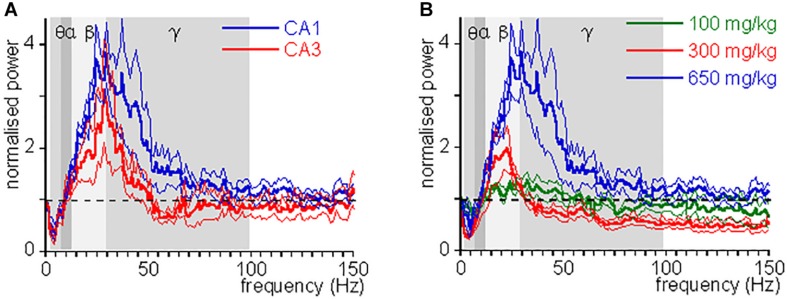
CH-induced change in power as function of frequency. **(A)** Oscillation power after a lethal dose of CH (650 mg/kg) administration, normalized to the oscillation power at baseline, as function of frequency, for CA1 (blue lines) and CA3 (red lines). Thick lines give mean, hair lines give s.e.m. Whereas changes in θ power and β power were similar in CA1 and CA3, the change in low γ power was typical for CA1. **(B)** CH-induced power changes at 100 mg/kg (green lines), 300 mg/kg (red lines), and 650 mg/kg (blue lines). Thick lines give mean, hair lines give s.e.m. Whereas changes in θ power and β power increase with dose, γ power first reduces and then increases with dose.

An CH overdose also increased β power in CA3 by 139.9 ± 41.8% [*Z*_(6)_ = −1.99, *P* = 0.046] and increased γ power by 20.7 ± 13.7% [*Z*_(6)_ = −1.99, *P* = 0.046], but strongly reduced θ power by 52.0 ± 17.0% [*Z*_(6)_ = −1.99, *P* = 0.046]. CH had no effect on α power [98.8 ± 15.6% of baseline, *Z*_(6)_ = −0.734, *P* = 0.463] (example in Figure 1D). In recordings from rats with a discernible high-frequency oscillation power peak, CH reduced the peak frequency decreased to 31.3 ± 4.5 Hz [*Z*_(6)_ = −2.02, *P* = 0.043]. Figure 2A gives the average CH-induced changes in power as function of frequency for eight rats in CA1 and ipsilateral CA3, which shows that, whereas changes in θ power and β power were similar in CA1 and CA3, the increase in γ power was typical for CA1. The increases in β power were mainly in the “upper β” (20–30 Hz) range and the increase in CA1 γ was limited to the low-frequency γ (30–55 Hz) range.

These observations indicate that a CH overdose causes a surge in high-frequency oscillations and a suppression of theta activity in the hippocampus.

### CH Changes Inter-Regional Coherence

**FIGURE 3:**
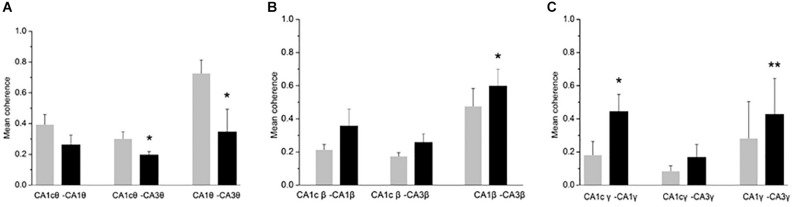
CH-induced increase in coherence between CA1 and CA3 areas. Inter-regional coherence was quantified between CA3 and CA1 ipsilateral, CA3 and CA1 contralateral and bilateral area CA1. **A:** Coherence in the θ band. Data are given as mean ± s.e.m. (Student’s paired *t*-test probability is indicated as: ^*^P < 0.05; ^∗∗^*P* < 0.01, *n* = 5). **(B)** Coherence in the β band. Details as in **(A)**. **(C)** Coherence in the low γ band (30–55 Hz). Details as in **(A)**.

Oscillatory activity is involved in the dynamic coupling of information transfer between anatomically connected areas (Colgin et al., 2009; Fries, 2015). To explore whether a CH overdose affects the inter-regional synchronization of oscillatory activity, we used a measure of inter-regional coherence at selected frequency bands. In five rats tested, inter-regional coherence was quantified between CA3 and ipsilateral CA1, CA3 and contralateral CA1 and CA1 bilateral, for the θ band, the β and the low-frequency γ band (where the CH-induced changes were most prominent). CH increased coherence in the β band between CA3 and ipsilateral CA1 (Figure 3B). CH increased coherence in the low γ band between bilateral CA1 areas and between CA3 and ipsilateral CA1 (Figure 3C). At the same time CH reduced coherence in the θ band between CA3 and contralateral CA1 areas and between CA3 and ipsilateral CA1 (Figure 3A). This suggests that CH increases synchronization of high-frequency oscillations across and between the hippocampi, whereas inter-regional theta synchronization reduced.

### Dose-Dependency of the Effect of CH on CA1 Oscillatory Activity

Chloral hydrate is used as a pediatric sedative safely up to 80 mg/kg (Avlonitou et al., 2011). To test whether and how CH affects oscillatory activity at different doses, we administered 100, 300, or 1000 mg/kg, while recording oscillatory activity in CA1. At 1000 mg/kg CH caused a rapid respiratory arrest and collapse of all oscillatory activity in four rats tested and only in one an increase in high-frequency oscillations could be observed before the collapse of oscillatory activity. Neither 100 mg/kg nor 300 mg/kg caused death. Figure 2B gives the average CH-induced power changes at different doses. At 100 mg/kg CH caused a small increase in β power, but had no consistent effect on power in other frequency bands (Table 1). Like with an overdose, 300 mg/kg CH reduced θ power and increased β power in CA1. However, in contrast with an overdose, 300 mg/kg CH decreased γ power (Table 1 and Figure 2B). The CH-induced enhancement of β power was dose-dependent [*F*_(2,27)_ = 4.42, *P* = 0.011], as was the suppression of θ power [*F*_(2,27)_ = 9.03, *P* = 0.001]. In contrast the dose-dependence of γ power [*F*_(2,27)_ = 3.65, *P* = 0.039] was due to the difference between the suppression at 300 mg/kg and the enhancement of γ oscillations at a lethal CH dose (Turkey *post hoc*, *P* = 0.054).

### The Effect of CH on the ECG and Breathing Rate

**FIGURE 4:**
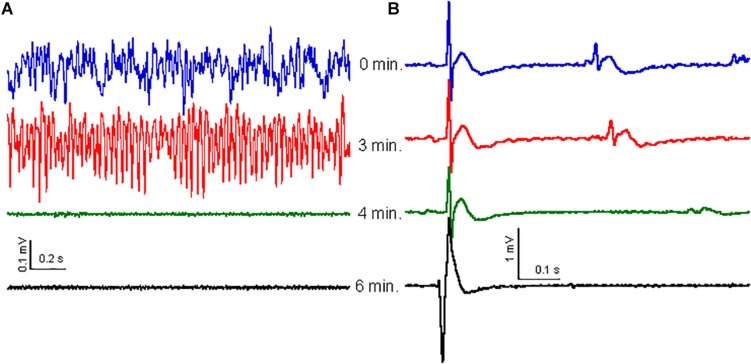
Effect of CH on oscillatory activity is not related to ECG. **(A)** Examples of oscillatory activity at 0 min (blue trace), 3 min (red trace), 4 min (just after collapse of oscillatory activity: green trace) and 6 min (black trace) after CH (650 mg/kg) administration. **(B)** ECG waveform average, over 30 s, zeroed at the peak of the QRS complex for the same times as in **(A)**. The ECG waveform did not change with the high-frequency surge or the oscillatory activity collapse, but widened and inversed and increased in amplitude 2–3 min after collapse of oscillatory activity, just before cardiac arrest.

The high-frequency oscillation surge observed in dying rats was only observed after cardiac arrest was established (Borjigin et al., 2013). It is therefore possible that CH causes the high-frequency oscillation surge as a consequence of cardiac arrest. To check for this, we recorded ECG simultaneously with the local field potentials in the hippocampus of five rats. Before CH (650 mg/kg) administration, the HR, calculated from the ECG, was 237 ± 22 beats per minute (bpm). After CH administration the HR reduced gradually to 187 ± 17 bpm [*t*_(4)_ = 4.03, *P* = 0.016] during the high-frequency oscillation surge and to 167 ± 17 bpm [*t*_(4)_ = 6.21, *P* = 0.003] during the collapse of oscillatory activity. The ECG waveform (zeroed at the QRS peak waveform) was stable throughout the high-frequency oscillation surge and collapse of oscillatory activity (Figure 4B). The QRS complex amplitude did not change during the high-frequency oscillation surge 94 ± 5% of baseline, [*t*_(4)_ = 1.32, *P* = 0.256]. Only after oscillatory activity in the brain had collapsed (Figure 4A), HR rapidly decreased to 41 ± 11 bpm [*t*_(4)_ = 16.42, *P* < 0.001], 2 min after the collapse. A heartbeat continued for several minutes, but the ECG waveform changed dramatically (Figure 4B), suggesting contractions that were not controlled by the normal conductive system and were likely to be inefficient.

Because the cardiac arrest occurred only after the high-frequency oscillation surge, the latter was not caused by cardiac arrest. The breathing rate was monitored by inspecting video recordings in three rats and dropped gradually soon after CH administration. At the time of the high-frequency oscillation surge breathing had dropped to 62 ± 2% of the baseline breathing rate [*t*_(2)_ = 4.49, *P* = 0.046] and breathing ceased completely just before the collapse of oscillatory activity, indicating that the high-frequency oscillation surge was associated with hypercapnia.

## Discussion

In anesthetized rats, death caused by an overdose of CH, induced a transient surge in high-frequency oscillations in the hippocampus and increased inter-regional coherence of high-frequency oscillations within and between the hippocampi. In contrast, CH-induced death was associated with suppressed theta oscillations and reduced inter-regional coherence of theta oscillations. The high-frequency oscillation surge was associated with a reduced breathing rate but occurred well ahead of the cardiac arrest following cessation of breathing and collapse of oscillatory activity.

### Potential Mechanisms Underlying the High-Frequency Oscillation Surge

High-frequency oscillations in CA3 partially drive oscillations in CA1 (Cardin et al., 2009). However, there were distinct differences between CH-induced changes in β power and γ power in CA1 and CA3 in the response to CH. Whereas CH reduced the peak frequency in CA3, it increased the peak frequency in CA1. This suggests that the increase in β power in both CA3 and CA1 is likely driven by an increase in β synchronization generated in the CA3 network, whereas the increase in γ synchronization at a fatal CH dose, is selective to CA1.

The CH-induced increase in β power and decrease of θ power already happened at sub-lethal doses and these effects increase with dose, suggesting a direct effect of CH on oscillatory activity, which is similar to the effect of other sedatives, like barbiturates (Fu et al., 2008) and benzodiazepines (van Lier et al., 2004) on oscillatory activity. The common mechanism of action is likely an increase in GABA receptor-mediated currents, because the active CH metabolite 2,2,2-trichloroethanol increases GABAergic currents in a way similar to barbiturates (Peoples and Weight, 1994). This causes a shift of the dominant high frequency to lower frequencies (Fisahn et al., 1998) and explains the increase in β power at the expense of γ power. This disruption of the careful interplay of θ, β and γ rhythms (Fries, 2015), shared amongst sedatives may therefore contribute to their sedative mechanism of action (Fu et al., 2008; Akeju and Brown, 2017).

Trichloroethanol can also hyperpolarize neurons by activation of two-pore potassium channels, which are assumed to contribute to the anesthetic effect of CH (Harinath and Sikdar, 2004). However, the net effect of a hyperpolarization on oscillatory activity is dependent on what type of cells these channels are expressed.

The surge in γ power and inter-regional γ band coherence with a fatal CH dose, contrasts with the γ power suppression of CH at sedative and anesthetic doses. Trichloroethanol inhibits NMDA receptor and kainate receptor activity (Peoples and Weight, 1998; Salous et al., 2009) by internalizing the receptors (LacKamp et al., 2009). Kainate receptor activation is used as a model to induce hippocampal γ oscillations *in vitro* (Hajos et al., 2000) and NMDA receptor antagonists like ketamine, are known to boost γ oscillations *in vivo* (Lazarewicz et al., 2010).

The two opposing effects of CH on γ band synchronization may explain why γ oscillations in CA1 show a bi-phasic dose-response relationship.

The death-related surge in γ band synchronization was similar to that observed in rats and humans after cardiac arrest (Auyong et al., 2010; Borjigin et al., 2013). In humans a CH overdose can cause ventricular tachycardia (Bowyer and Glasser, 1980). However, in our hands, a CH overdose caused mild bradycardia and the high-frequency oscillation surge happened well before substantial cardiovascular impairment. Our results fit therefore better with the high-frequency “end-of-life electroencephalographic surge” observed in dying patients that occurred minutes before loss of blood pressure (Chawla et al., 2017). This high-frequency oscillation surge may, instead, result from the observed reduction in breathing rate and the consequently developing hypercapnia. CH is known to cause respiratory depression at doses used for sedation (Ganigara et al., 2019) and in rats at anesthetic doses (Field et al., 1993), which is likely to be exaggerated under urethane anesthesia. Hypercapnia develops quickly and the resulting acidosis increases activity of the gap junction-forming connexin 36 (Gonzalez-Nieto et al., 2008), which is crucially involved with hippocampal γ oscillations (Buhl et al., 2003). Increased γ oscillations have also been observed with spreading depression, which also relied on increased gap junction activity (Herreras et al., 1994). However, in contrast to what is expected from spreading depression, we did not observe any systematic difference in the time course of the high-frequency oscillation surge recorded at different locations.

### Potential Mechanism Underlying the Collapse of Oscillatory Activity

The hypoxia that develops after cessation of breathing is likely to suppress oscillatory activity, which was observed with partial hypoxia, while maintaining pH in an *in vitro* γ oscillation model (Pietersen et al., 2009). The collapse of all oscillatory activity simultaneously at all locations is similar to the non-spreading suppression, which was observed in both humans and rats after circulation stops, coinciding with the delayed drop in pO_2_ after cessation of breathing (Dreier et al., 2018). This collapse reflects a shutdown of neuronal activity by hyperpolarization due to opening of calcium-dependent potassium channels (Revah et al., 2016).

### Functional Implications of the High-Frequency Oscillation Surge

Chloral hydrate increased β power and reduced θ power in both areas CA1 and CA3 already at sub-lethal doses. β oscillations are traditionally associated with sensorimotor functions (Kilavik et al., 2013), but are also implicated in top-down processing and long-range communication (Fries, 2015; Akeju and Brown, 2017; Spitzer and Haegens, 2017). Increases in the “upper” β range (20–30 Hz), typical for the fatal CH dose, have been implicated with interactions between attention and emotion systems (Marco-Pallares et al., 2015).

A fatal CH dose caused, in addition to the changes in θ power and β power, a transient increase in γ power in CA1, an area, especially involved in contextual memory retrieval (Ji and Maren, 2008; Dimsdale-Zucker et al., 2018). Increases in power and coherence of γ oscillations have been associated with episodic memory acquisition and retrieval (Hanslmayr et al., 2016). Interestingly, the increase in γ power was limited to the low-frequency γ range (30–55 Hz) in CA1, which is driven by CA3 and plays a role in memory retrieval (Colgin et al., 2009). The θ oscillation normally regulates the alternation between memory acquisition and retrieval (Colgin et al., 2009). The careful interplay of θ, β and γ rhythms render neuronal communication effective, precise, and selective (Fries, 2015). It is possible that the CH-induced suppression of θ oscillations favors low-frequency γ oscillations and, aided by increased “upper” β range activity, facilitates episodic memory retrieval at the expense of perception of reality. A similar suppression of θ power and increase in γ power is similar to the effect of the psychotomimetic ketamine (Lazarewicz et al., 2010). It is tempting to speculate that this brief state of distorted oscillatory activity that may facilitate episodic memory retrieval and/or distort perception, just before collapse of neuronal activity due to hypoxia, underlies the NDE reported in patients after cardiac arrest (Auyong et al., 2010).

Chloral hydrate overdose can cause ventricular tachycardia in humans (Bowyer and Glasser, 1980). However, because in our hands, CH overdose caused mild bradycardia and the high-frequency oscillation surge happened well before substantial cardiovascular impairment, our results fit better with the high-frequency “end-of-life electroencephalographic surge” observed in dying patients that occurred minutes before loss of blood pressure (Chawla et al., 2017). Our results suggest that the high-frequency oscillation surge associated with NDE is not dependent on cardiac arrest, but is a more general response to a failing support for metabolic brain function.

## Data Availability

The datasets generated for this manuscript are available on request to the corresponding authors, or directly at zyj-103@163.com.

## Ethics Statement

This study was carried out in accordance with the recommendations of animal ethics and administrative council of Xinxiang Medical University. The protocol was approved by the Xinxiang Medical University.

## Author Contributions

YZ performed the experiments, analyzed the data, and wrote the manuscript. ZL and JZ performed the experiments. ZZ analyzed the data. HZ supervised the study and analyzed the data. MV supervised the study, analyzed the data, and wrote and revised the manuscript. CL designed the study, analyzed the data, and wrote and revised the manuscript.

## Conflict of Interest Statement

The authors declare that the research was conducted in the absence of any commercial or financial relationships that could be construed as a potential conflict of interest.
